# Outline of Lecture

**Published:** 1877-01

**Authors:** 


AMERICAN JOURNAL

OF

DENTAL SCIENCE.

Vol. X. THIRD SERIES—JANUARY, 1877. No. 9.

### ARTICLE I.

### *Outline of Lecture, December 1st, 1876.*

BY PROF. HODGKIN.

GENTLEMEN.—We have taken our impression and dismissed our patient. The subsequent treatment of this impression is influenced and modified by the character of the impression material and the style of work we may decide upon. These modifications we shall take up in detail.

Our patient is gone—a few words as to our treatment of his case and himself, and of the relations we sustain, or should sustain, professionally to him.

*As to our deportment*: it should be gentlemanly. Looseness, or even freedom toward those who consult us is offensive; they come to get our advice and work, not a history of our family grievances and our office practice. Don't bore your patient needlessly with your overflowing conversation. If talking is to be done, let it be instructive to him in your science. You may hear very judiciously advice—but don't overflow; you know the effect of an overdose therapeutically.

Don't hurt your patient needlessly. Long finger-nails, rings with seals, and such things often cause pain. Cut the nails short and see that they are scrupulously clean. Your young and beautiful patient may combine beauty and the beast by neglecting this care of her own person, but she will not tolerate it in her dentist. A little ammonia or alcohol on the corner of a napkin, will remove difficult stains here and elsewhere about the hands. Bicarb soda is useful where we have been handling flasks.

Be neat in your dress. If possible; wear a clean linen coat or jacket whilst operating; it produces a pleasant impression and looks nicely, and looks go a long way as you well know. Most people form their opinions of you from very hasty and inadequate observations, and we know how lasting is a first impression. Be scrupulously nice with your teeth. Your patient will value his own more highly if he sees your's carefully attended to.

*The breath* is a subject of very serious moment. A dentist with a fœtid breath is an object of disgust, and has no right to afflict others with his chronic offensiveness; it is intolerable and will cause us serious loss, unless very great talents, which we may find later that we do not possess, offset it. Ascertain the cause of this fœtor and remove it; stumps of teeth, indigestion, uncleanliness, &c.,—search the ground carefully, for it is a grave evil. A little soap on the tooth-brush and a vigorous scrubbing of the back part of the tongue, goes far to annihilate this annoyance. Avoid whiskey and tobacco. Close observation of those who use these things will teach you why. Polish your instruments and cups. Be neat, tidy, to a fault.

We look at our impression a moment again. It is the foundation of our work, and on its accuracy or want of accuracy depends largely our success or failure. If we have made a philosophic selection of our impression material, used a proper cup and conducted these various processes properly, we will doubtless not be disappointed. Certainly no pains should be spared and no cost of time or money spared which will give us complete accuracy. But if we feel that any improvement could be made by trying again, save the first impression (for obvious reasons) and take another. Let nothing pass, as is so commonly done with other kinds of work, with the flippant, lazy remark : "Not very good, but I guess it will do." No "pretty nearly right" work must be ours. It ought to be just right or not at all.

Bear this steadily in mind, that a workman's reputation rests on his poorest, not on his best work ; and the world will value you not for what you know, but for what you can do. It is of little avail for you to discourse learnedly about our science, if your pieces fit badly or are of slovenly construction.

I feel as though it were lowering myself and you, to urge honesty. But we lose sight of that truer, nobler idea of honesty, in thinking of that mean and stingy puritanic proverb, "that it (honesty) is the best policy." I don't mean that sort of poor and pitiful honesty which is thus enforced; but that higher sentiment which scorns the mean and low, which does right not because it will *pay*, but because it is *right.*

It is no injustice to us to work, and in working to expect a reward ; it is proper that we should be paid and paid well, for we expect and are expected to give our best efforts. But woe to the man who makes this his "be all and end all." If his conception of what is due his profession, his patients and himself, rises no higher than this, it were better for him, that is professionally, "that a mill stone were hung about his neck," &c. Just in proportion as we can isolate ourselves from the idea of fees and rewards, just in proportion as we do this "for the work's sake," just in that proportion will we rise to the true dignity of our calling. I am sorry for that man who has more enjoyment in the prospect of a fee than in the knowledge that his work is esthetically beautiful, natural and serviceable. He is on a road downward, and not upward ; his profession need not expect elevation at his hands. It is only benefitted and elevated by the man who is willing, nay, anxious to spend and be spent in its service ; to give it his best thoughts and efforts. How the character of such a man as Chapin A. Harris, looms up grandly before us when compared with some one of our modern "steam dentists," whose knowledge "begins with vulcanizable gum and ends with a hard rubber plate."

Unless we love our profession, it is useless for us to think of advancing and elevating its condition. Such a sentiment as this, for example, which I have heard in the mouth of a practicing dentist, is a reproach to the cause, and a confession of his utter unfitness for his chosen profession : "I thought dentistry paid pretty well, was the reason I thought of being a dentist, and if I could find some more profitable work I would give up practice."

No, gentlemeu, let me adjure you as those to whom your preceptors look to honor and exemplify their teachings ; let me urge you as those who must help to move the world, if it move at all, *love your work ;* be enamored of your profession, enter heartily into its study as into a chosen and lofty calling, one to which you may give the best energies of your nature with a feeling that it will be elevating and ennobling, and in so doing, you will find a reward above computing in dollars and cents.

Our patients put themselves in our hands. No matter what selfish view they may take of the subject—no matter how *they* may feel about its being a question of pay with you,—to *you*, *yourself*, if you are a true man, it can assume but one phase : *what is the very best I can do for this person who has put himself in my hands.* If I fall short of my ideal, if I allow myself to falter in what I feel is right—if in a word, I fail to do my duty wholly, fully, utterly, in its entirety—have I any right to the position I occupy ?

I know how often this question assumes a shape like this in the struggle between duty and interest; how we may be tempted to put the case thus, and stifle the monitions of our professional conscience by saying mentally : " This is as good work as I can afford to do for the pay." You cannot afford to do it badly at any price. Or we may perhaps truthfully enough say : " It is better done any way than my neighbor would have done it." We have no right to make such compromises.

The path of duty only is the path of safety for us. Our stern, inflexible duty to our patients, uncompromised by any reflection on our neighbors' capacity or honesty, and uninfluenced by mercenary motives.

If we fail to rise to this elevated standpoint, if we do not feel in our hearts and consciences' that our patients welfare is the goal for which we strive, if we make the mere question of *fees* the chief and controlling one with us, our future cannot be a success in so far as the advancement of our calling is concerned, and we had better not be dentists. If on the other hand, we are earnestly striving and are fully resolved that we will give our best efforts to our work irrespective of fees and rewards; the reward shall surely be ours in a satisfaction greater than that of fees. There is a reward more valuable than dollars and cents ; there is remuneration more satisfactory than a fee; it is found in the conscientious discharge of our plain duty. Nor is this reward, as some have taught to be found wholly hereafter, in a future state, but it is instant in its reception and abiding in its character.

---

